# Analysis of electrode performance on amplitude integrated electroencephalography in neonates: evaluation of a new electrode aCUP-E vs. liquid gel electrodes

**DOI:** 10.3389/fped.2024.1452862

**Published:** 2024-10-03

**Authors:** Albert Fabregat-Sanjuan, Ángel Rodríguez-Ballabriga, Agnès Rigo-Vidal, Rosa Pàmies-Vilà, Susana Larrosa-Capaces, Vanesa Rius-Costa, Vicenç Pascual-Rubio

**Affiliations:** ^1^ FUNCMAT, Mechanical Engineering Department, Universitat Rovira i Virgili, Tarragona, Spain; ^2^ NeuroÈpia, Institut d’Investigació Sanitària Pere Virgili, Clinical Neurophysiology Department, Hospital Sant Joan de Reus, Reus, Spain; ^3^ BIOMEC, Mechanical Engineering Department, Universitat Politècnica de Catalunya, Barcelona, Spain

**Keywords:** aEEG, impedance, neonates, electrodes, neonatal intensive care unit, aCUP-E, preterm, signal artifacts

## Abstract

**Background:**

Neonatologists and clinical neurophysiologists face challenges with the current electrodes used for long-duration amplitude-integrated electroencephalography (aEEG) in neonatal intensive care units (NICU), limiting the capacity to diagnose brain damage.

**Objectives:**

The objectives of this study were to develop methods for comparing the performance of different electrodes to be used in aEEG. The comparison was done between a newly designed neonate-specific electrode, aCUP-E, with commercial liquid gel electrodes used in amplitude-integrated electroencephalography (aEEG). The comparison included impedance stability, electrode survival, recording quality, usability, and satisfaction of NICU staff.

**Methods:**

aEEG recordings with bipolar montage was used, with one hemisphere fitted with commercial electrodes and the other with aCUP-E electrodes, alternated among subjects. Continuous impedance and raw EEG data were collected over a minimum of 24 h, and signal processing was performed using Python and MATLAB.

**Main results:**

aCUP-E electrodes demonstrated superior performance, including: Increased impedance stability and electrode survival, enhanced recording quality with fewer artifacts, high correlation in signal capture between electrodes during optimal brain activity segments, higher signal-to-noise ratio (SNR) across varying impedance levels, greater staff satisfaction and ease of use. Moreover, Kaplan-Meier curves indicated a higher survival rate for aCUP-E electrodes over 24 h compared to commercial electrodes. Impedance variability analysis showed statistically significant stability improvements for aCUP-E.

**Conclusion:**

aCUP-E electrodes outperform commercial liquid gel electrodes in impedance stability, electrode survival, and recording quality. These results suggest that aCUP-E electrodes could significantly enhance aEEG utilization in diagnosing and treating neonatal brain conditions in NICUs. Future improvements to the aCUP-E electrode may further reduce artifacts and increase electrode longevity, potentially leading to a significant improvement in neonatal brain monitoring by means of aEEG.

## 1 Introduction

In the forefront of neonatal care, significant progress has been achieved. However, assessing neonatal brain function comprehensively remains a challenge due to the complexities of neonatal neurophysiology and the subtleties of early brain development characteristics. Within Neonatal Intensive Care Units (NICUs), neurological pathologies emerge as notable factors, comprising roughly a quarter of admissions (1, 2). Both neonatologists and clinical neurophysiologists concur on the imperative to enhance existing technologies for more accurate recording of brain activity, thereby enhancing the overall care provided to critically ill neonates, and facilitating early interventions aimed at minimizing potential brain damage (3, 4).

Amplitude-integrated EEG (aEEG) is considered one of the most suitable methods for continuous monitoring of brain function in neonates and is increasingly used in NICUs (5). Its characteristic processing of the EEG signal makes it possible to show the cycling of the compacted EEG trace, which is a very important feature when assessing the neurodevelopmental process of the newborn (6). Its main technical advantages are based on the fact that few electrodes are used, which allow quick setting and a global visual access to the newborn's head, to be able, for example, to explore the fontanelles or to have access to the epicranial veins. Because the more frequent aEEG uses only 4 recording electrodes placed in centroparietal regions to record brain activity, the recording has less sensitivity than EEG for detecting focal damage but allows efficient monitoring of overall brain function and neurodevelopment facilitating long-term surveillance of cerebral function (7). However, it still has not yet been completely deployed in clinics as a gold standard because there is a controversy among clinical professionals about its difficult interpretation (4), which requires specialized training; as well as some technical limitations to overcome (8). Current cerebral function monitors (CFM) have good usability and are able to correctly show the signal as well as have particular algorithms for pattern recognition. However, the main technical limitations are due to surface electrodes because of detachments and gel drying. Such electrode issues can generate artifacts in the signal, which significantly affect the accurate interpretation of the aEEG data. One of the purposes of the aEEG is to quickly detect encephalopathies and/or seizures in order to treat them early, since time is a decisive factor, and thus minimize their potential damage to the brain. Since there are currently highly specific treatments such as hypothermia therapy or the administration of antiepileptic drugs, the methods must be precise enough not to give rise to any false positives, as these treatments can cause significant side effects (9, 10).

A non-trivial difficulty in having a good aEEG recording is to obtain a good quality and artifact-free recording of the brain bioelectrical signal. Therefore, the acquired signal is highly dependent on having a very good impedance during a long period of time (at least 24 h). Failure to do so may result in impedance variability that is strongly related to increased bioelectrical noise preventing correct assessment of the record. Or even excessively high or changing impedance can cause aEEG patterns similar to those seen in seizures and thus lead to one of the most dreaded errors in interpretation, which is false positives. The latter is one of the main concerns of clinicians when using aEEG technique (11).

Consequently, although in recent years major technological improvements have focused in CFM that allows better signal processing, raw EEG signal availability, and even seizures detection programs (12) and established EEG patterns (13), electrodes are a key factor for this technique and some efforts are being made to find an optimal solution that adapts to neonates (11). Subdermal needle electrodes, despite allowing low-impedance recording, are not recommended in newborns because they are invasive, especially in preterm, as they may have alterations in coagulability. Moreover, gold cup electrodes, being rigid and requiring external pressure to be held, can cause damage to the newborn's fragile scalp. Also, the colloid used to stick the electrodes in adults is not recommended in newborns (14). Hydrogel electrodes show improved safety and probability of survival compared to gold cup electrodes but require numerous re-placements (15).

For this reason, the most suitable and commonly used electrodes in the clinical practice are self-adhesive with liquid gel (15). This is because of their quick placement and non-invasiveness, which is crucial for the neonate's fragile skin. Despite these improvements in the ease of use, there are still several challenges to overcome, such as impedance level, impedance stability and the survival time, which are known to have an influence on the quality of the recording (16, 17).

A proper electrode that meets all the specific needs could help improve the acquired signal, reducing artifacts, and helping to broaden the use of aEEG to be used as a standard tool for brain monitoring in NICUs.

## 2 Methods

This work was performed in the framework of a clinical trial that was being conducted at *Hospital Universitari Sant Joan de Reus (HUSJR)* and that was approved by Ethical committee of Institut d’Investigació Sanitària Pere Virgili (IISPV) with reference number 032/2021 and also has the authorization from the Spanish Agency of drugs and medical devices (AEMPS) with reference 918/21/EC-R.

The main objective was to compare the new electrode, aCUP-E (advanced cup electrode), specifically designed by this research group to perform amplitude-integrated electroencephalography (aEEG) in the neonatal intensive care unit (NICU); with the liquid gel electrodes currently used in *HUSJR* and recommended by the centres with more experience in aEEG (15). These commercial electrodes are Neuroline 720 from Ambu®, not specifically designed for neonates.

**Figure 1:**
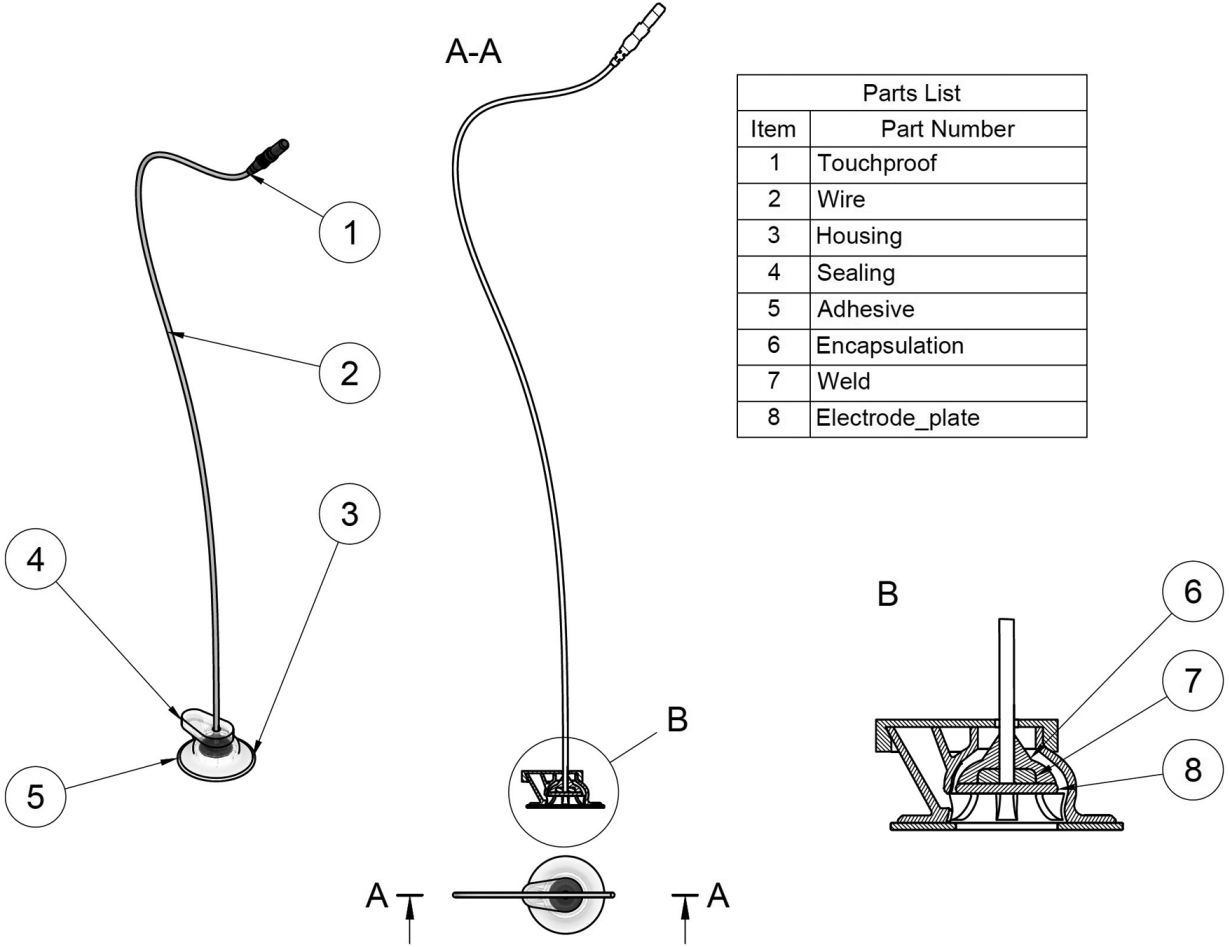
aCUP-E electrode. (1) Universal connector, able to connect to all EEG devices. (2) Short wire, to facilitate disconnection with monitor for improved newborn handling. (3) Housing, the main part that allows safe gel injection/replacement and electrode plate fixation. (4) Sealing, cover to reduce electroconductive gel drying. (5) Adhesive for delicate skins, long recordings, and good adherence. (6) Encapsulation to insulate weld from electroconductive gel. (7) Weld between electrode plate and wire. (8) Ag/AgCl Electrode plate.

The driving force behind the aCUP-E development was to overcome the recognized shortcomings that commercial electrodes present when applied to long-lasting recordings on neonates (primarily big dimensions and poor adhesion). It is flexible and translucid, with a firm and safe adhesion for the fragile skin of the neonatal scalp. Its use is indicated for cases in which long studies are required, as it shows good adhesion, easy replacement of the electroconductive gel without detaching the electrode form the skin, keeps a stable impedance, permits skin irritation checking because its translucency and allows a comfortable manipulation of the newborn by the healthcare personnel through a short wire connection (Figure 1).

The starting hypothesis of the study is that the new electrodes have the same safety compared to liquid gel electrodes (commercial), having the advantages of a better adhesion, a more stable impedance (reducing the number of artefacts), a more comfortable handling of the baby by the healthcare personnel and longer recordings without removing the electrode.

### 2.1 Electrode placement

A bipolar montage comprising 5 electrodes was employed to obtain one signal from each hemisphere. Electrode positions were determined according to the International 10–20 system, with C4 and P4 utilized for the right hemisphere, C3 and P3 for the left hemisphere, and the neutral electrode is positioned anterior in the midline. This arrangement facilitated the recording of one hemisphere with liquid gel (commercial) electrodes and the other with aCUP-E electrodes. Comparison between hemispheres was feasible due to the synchronous development of both hemispheres in healthy neonates, notwithstanding variations in post-conceptional ages (PCA) (18).

**Figure 2:**
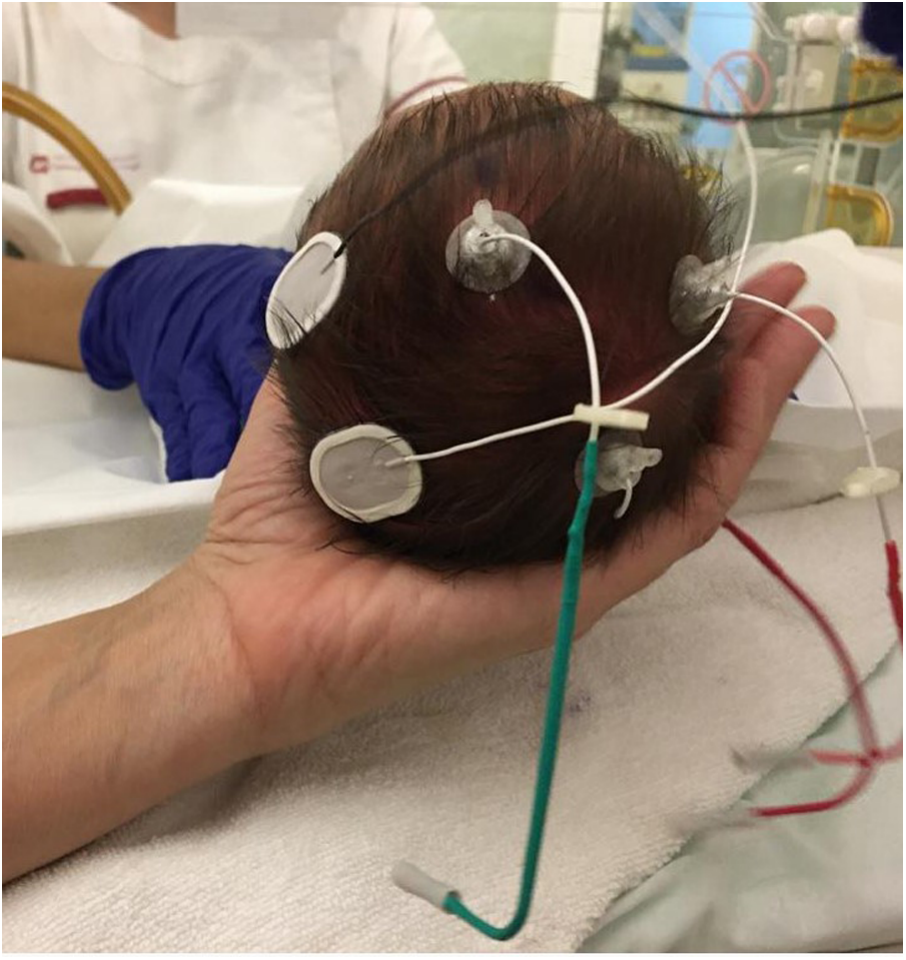
Electrode montage used in the clinical research in which two electrodes of each type are used to record the brain activity of one hemisphere. The subject is in supine position. (Left hemisphere) liquid gel electrodes, (Right hemisphere) aCUP-E and (central electrode-reference) aCUP-E (Case 2).

Both types of electrodes were concurrently employed during recording sessions, with commercial electrodes utilized on one hemisphere and aCUP-E electrodes on the other, alternated among subjects. The reference electrode utilized was an aCUP-E electrode. Figure 2 illustrates the electrode placement on the neonatal scalp.

Preparation of the neonate's scalp was undertaken as standard procedure, prior to electrode attachment to optimize transmission of electrical activity by reducing impedance and enhancing electrode adhesion. This preparation involved identifying precise electrode positions according to the International 10–20 system and cleaning the corresponding scalp regions. Commercial electrodes did not require additional electroconductive gel as they were pre-embedded with liquid gel during manufacturing. Conversely, for aCUP-E electrodes, gel application was necessary after placement into the specified aperture and replenished every 8 h^1^.

Following electrode placement, connections were established with the aEEG device (CFM Olympic Brainz Monitor, Natus Medical Inc.), and recordings were conducted for a minimum duration of 24 h.

### 2.2 Data acquisition

**Figure 3:**
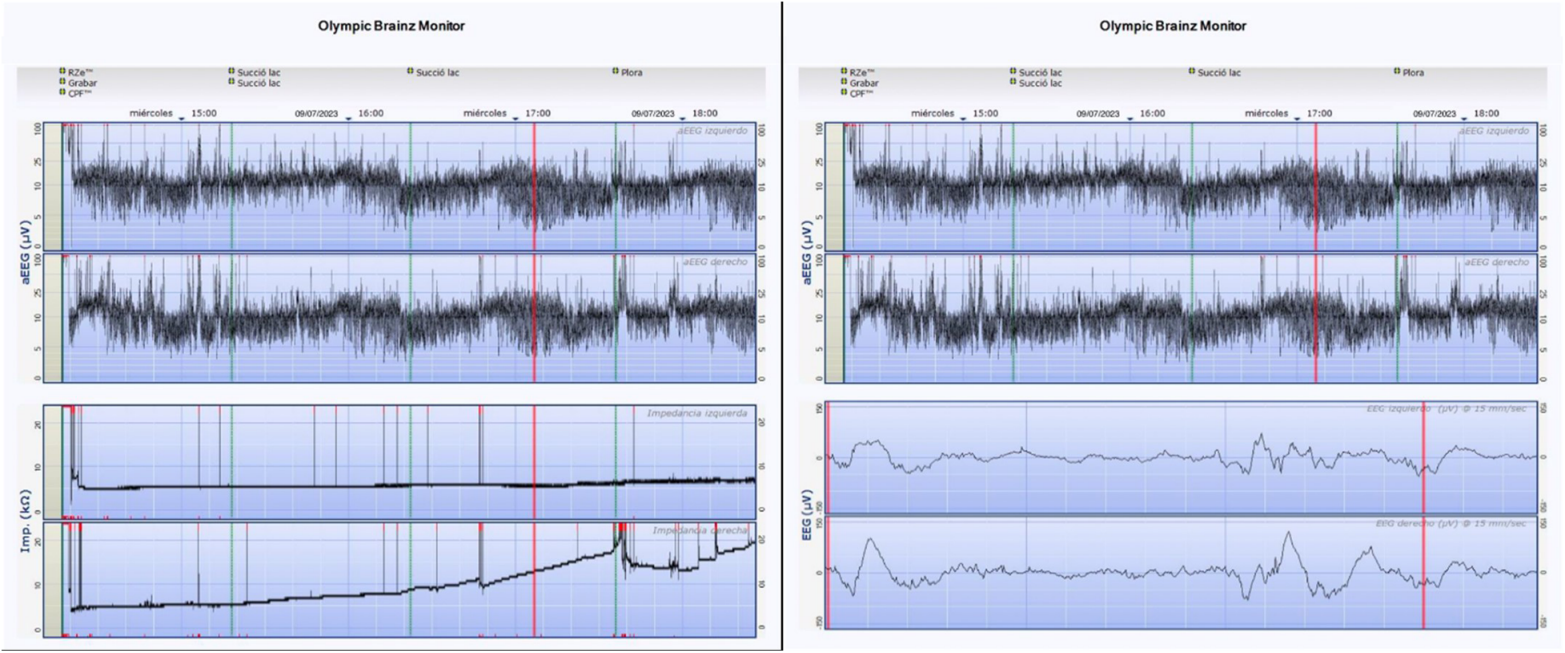
Example of the CFM screen with aCUP-E electrodes on the left hemisphere and liquid gel electrodes on the right hemisphere. The two visualization possibilities that offers de CFM are shown: the aEEG signal with the impedance and the aEEG signal with the raw EEG signal.

The utilized monitor (*CFM Olympic Brainz Monitor*) enabled recording of raw EEG data with a bipolar montage, continuous impedance monitoring (without interrupting recording), and aEEG, which was consistently displayed in the upper portion of the screen. Below this display, users could readily switch between the raw EEG and impedance signals using an on-screen button interface. Figure 3 shows an example of the CFM screen monitor where aEEG, impedance and raw EEG can be seen.

However, only files containing raw EEG and continuous impedance data were exportable from the monitor. These datasets were retrieved in European Data Format (EDF) and thereafter transformed into CSV (Comma-Separated Value) format utilizing the Python MNE package, selected for its adept management of EDF files. Subsequently, signal processing was conducted using MATLAB from *The MathWorks, Inc. (2022).* Details regarding the specific files of interest and their corresponding sampling frequencies are provided in Table 1.

**Table 1:** Characteristics of the files of interest extracted from the monitor for each acquisition, together with the electrodes (channels) that record that signal.

File name	Channel	Sampling frequency (Hz)
LeftEeg	C3-P3 bipolar signal	200
RightEeg	C4-P4 bipolar signal	200
LogicalImpedanceC3	C3 impedance	100
LogicalImpedanceC4	C4 impedance	100
LogicalImpedanceP3	P3 impedance	100
LogicalImpedanceP4	P4 impedance	100

Finally, after each recording, the involved nurses filled a survey regarding ease of placement, removal, and convenience; the comfort in handling the newborn by healthcare personnel and the safety of the newborn's skin.

### 2.3 Reproduction of clinical aEEG signal

Companies that market the brain function monitors consider the processing of aEEG to be part of their intellectual property. This does not allow researchers to directly obtain the aEEG recording or to know the details of the EEG signal processing used, which makes difficult for clinicians and researchers to choose between different brands, because no aEEG standard has been established so far (19).

Due to proprietary constraints imposed by companies marketing brain function monitors, direct access to aEEG recordings or details of EEG signal processing utilized are inaccessible to researchers. Raw EEG signals extracted from the monitor were processed to obtain aEEG data. A graphical interface was developed to allow interactive visualization of aEEG, together with the option to store variables for mathematical analysis. This approach aims to replicate the monitoring capabilities of clinicians on any computer, necessitating the interface to mirror key characteristics of medical devices. The code for aEEG signal extraction was adapted from the Washington University Neonatal EEG Analysis Toolbox (WU-NEAT, 2020) (20), predominantly based on works by Zhang et al. (21) and Chen C. et al. (22).

**Figure 4:**
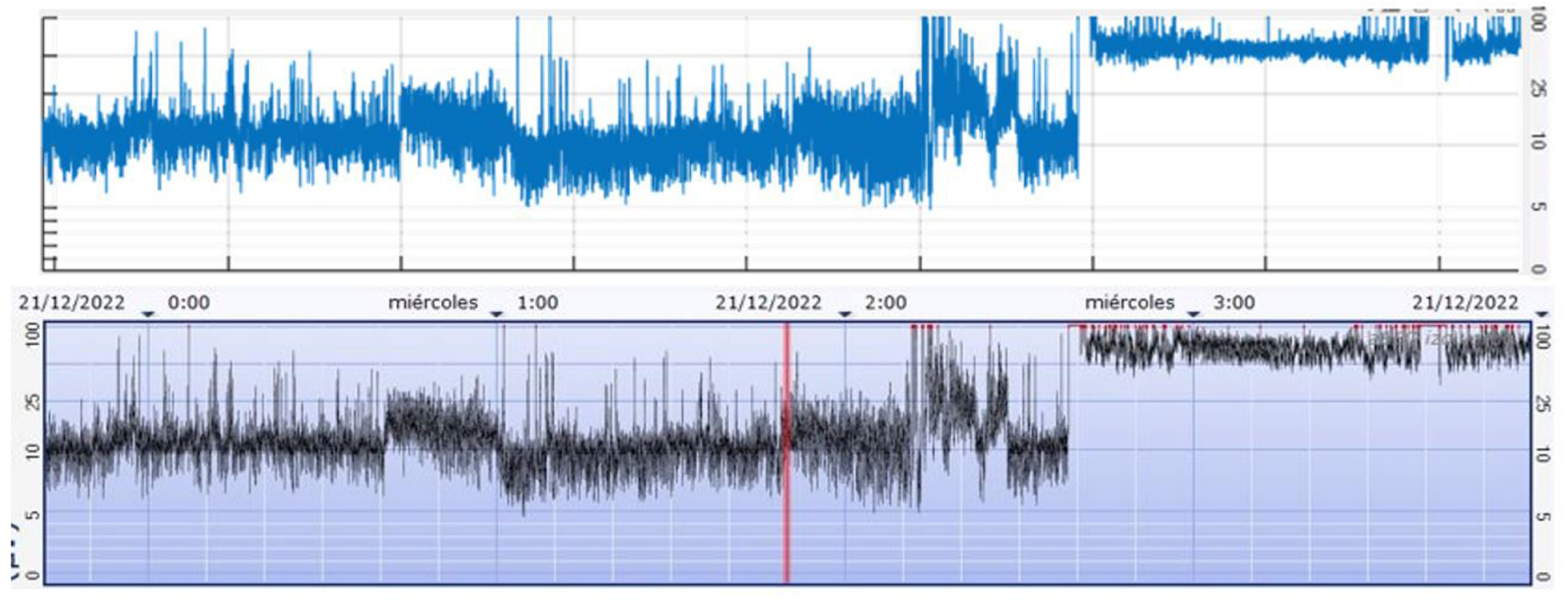
Case 1. Fragment of aEEG recording. (Above) Signal from the *CFM Olympic Brainz Monitor*, (Bottom) Digitally obtained signal.

The processing algorithm involves an asymmetric filter to compensate for skull attenuation at different frequencies, artifact elimination (focused on the 2–15 Hz range of interest in aEEG), signal rectification (absolute value), envelope detection (Butterworth low-pass filter), and semi-logarithmic scaling (*y*-axis) with a predefined ratio of 6 cm/h (*x*-axis). Adjustment of parameters (cut-off frequencies, filter orders, display proportions) was performed to ensure the obtained signal closely matched that of the clinically utilized monitor. Statistical comparison between the two signals was unfeasible due to the inability to extract aEEG signals directly from the medical device. Hence, a visual comparison was conducted between screenshots from the monitor and the created user interface (UI), with clinicians observing no discernible differences for diagnostic purposes (Figure 4).

**Figure 5:**
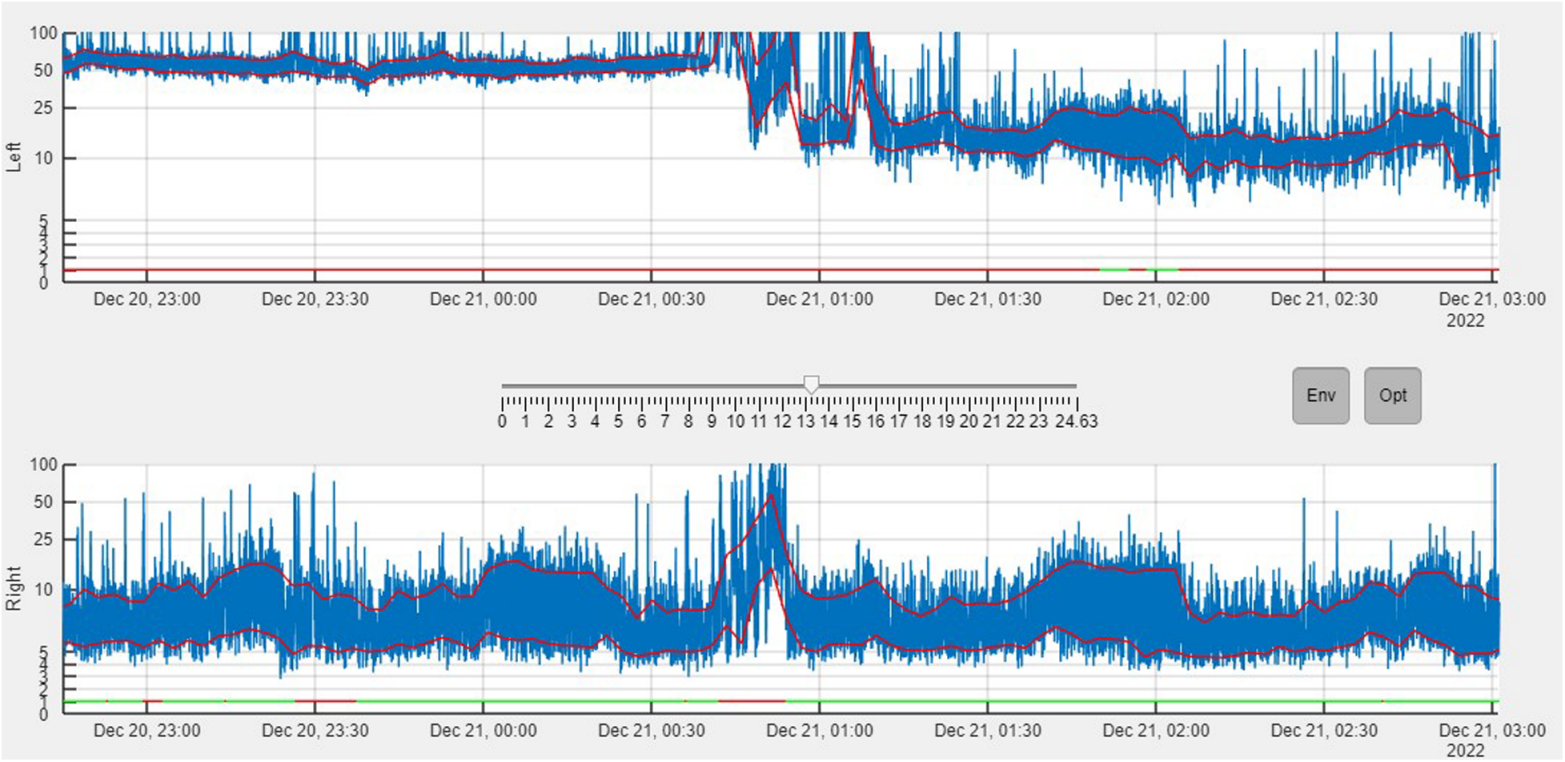
Ui program built in *MATLAB*, simulating an aEEG monitor. The aEEG (blue), the envelope (red line) and the parts where non-cerebral brain activity has been detected (straight line at the bottom of each hemisphere. Green: Cerebral activity, Red: Artifacts detected) can been seen. The signal in the top is the left hemisphere (commercial electrodes), while the one in the bottom is the right hemisphere (aCUP-E) (Case1). Button *Env* enables the margins while *Opt* enables the optimal/non-optimal line.

The upper and lower margins, as well as non-optimal brain activity (likely not coming from the brain) have also been processed and represented in the UI, allowing more signal features to be seen than the monitor allows (Figure 5).

To detect optimal brain activity in healthy newborns, two neurophysiological criteria have been proposed: EEG waves higher than 150 *μ*V and longer than two seconds wavelength are unlikely to be from brain activity, and, that peak-to-peak amplitudes close to 0 in aEEG are due to other factors (e.g., salt bridge).

### 2.4 Parameters analysed for performance comparison

Liquid gel electrodes are the best and safest option for aEEG (15) so new methods must be compared against them before being deemed suitable. The first difficulty is to agree on the evaluation procedure. In the literature, reports of performance have been conducted with non-homogenous methodologies, so that their results cannot easily be compared: Frequency response, impedance spectroscopy, EEG rhythm and evoked potentials comparison (23–25), usability, and staff satisfaction (15), durability and skin safety issues (14, 15) or SNR and proportion of artifacts (24). As a result, the following analysis is based on different relevant parameters found in the literature.

#### 2.4.1 Optimal and non-optimal brain activity

To ensure a valid comparison of interhemispheric electrode capture, it is imperative to select a time frame where both electrodes capture the signal without artifacts and maintain low and stable impedances. Failure to meet these criteria may introduce confounding factors that could influence the observed differences in aEEG signals. Therefore, the parameter selection is based on neurophysiological criteria (*2.3 Reproduction of the aEEG signal)* to assess how effectively both electrodes capture the signal, ensuring a minimum recording duration of at least 2 h for optimal comparison. Recordings failing to meet this requirement are excluded from further signal analysis, as insufficient time is available to conduct amplitude analysis (same-time different location).

#### 2.4.2 Reliable signal capture (bland-altman and SNR susceptibility)

Consistency in signal capture between electrodes is essential for clinicians to rely on new aCUP-E electrodes. To evaluate this consistency, Bland-Altman analysis is a suitable option (26). Adapting it to this specific case, the analysis is confined to regions identified in the previous section (optimal/non-optimal brain activity), where both electrodes should exhibit high correlation. Failure to meet this criterion warrants further evaluation of aCUP-E electrodes. Comparison of the signal capture in non-optimal time frames would be not correct, since many other factors not depending on the electrode would influence the signal.

Furthermore, power around the line noise frequency tends to increase with rising electrode impedance values. Various studies have explored the impact of 50 Hz noise power across different impedance ranges (23), and used SNR (Signal-to-noise ratio) as a measure of signal quality (24). Higher and more stable SNR would allow for a more accurate postprocessing (27).

Leveraging the continuous monitoring capabilities of the employed device, an investigation is conducted to explore the susceptibility of the SNR within specific impedance values. This experiment aims to determine if both electrodes behave similarly when examining frequency components collected by them.

This frequential analysis is performed in the framework of aEEG, hence the frequencies between 2 and 15 Hz will be considered the signal. For the noise, in EEG signals, we have different sources of noise (cardiac pulse, muscle contraction, electromagnetic interferences…). However, mains Hum noise (50 Hz) is the one that is more related with impedance and, therefore, it will be used for this analysis. Equation 1 shows how the SNR has been computed.

(1) $$SNR=10*\mathrm{lo}g_{10}\left(\frac{\mathrm{PowerSigna}l_{2-15Hz}\;(\mu V^{2})}{PowerNoise_{50\mathrm{Hz}}\;(\mu V^{2})}\right)\;(\mathrm{dB})$$

Thus, to enhance robustness, an evaluation of electrode behavior across varying impedance levels is imperative. To this end, a susceptibility analysis was conducted, assessing signal-to-noise ratio (SNR) for one-minute segments to characterize how SNR varies with impedance levels over all the recordings. This time frame was selected to ensure adequate frequency resolution and stable impedance.

#### 2.4.3 Electrode survival computation and criteria

The time at which each electrode ceases to function is computed using continuous impedance monitoring provided by the monitor. A cessation of functioning (e.g., detachment or gel drying) is assumed when an electrode exceeds 20 kΩ for at least 15 min, surpassing the average time taken by nurses to provide care. Upon impedance decrease, it is assumed the electrode has been manipulated or replaced, and the count restarts for the new electrode.

Additionally, based on El Ters et al. (14) analysis on electrode survival, all electrodes are considered to last at least 45 min. Detachments before this time are attributed to procedural errors during adherence and are excluded from analysis. These assumptions inform the construction of the algorithm.

Kaplan-Meier curves are employed to estimate the survival function and accommodate censored data (28). Only the first 24 h are considered in this analysis to ensure consistency, as recordings of varying lengths may affect probability function approximation. Moreover, cases where proper electrode adhesion was not initially achieved were excluded from this analysis. This exclusion was warranted as the cessation of functioning was attributed not to the electrodes themselves, but rather to the preparation of the recording. Therefore, cases 8 and 9 were excluded from the following survival analysis due to their poor impedance performance in all positions, irrespective of electrode type. Manipulations and breaches of protocol are not considered for either electrode type.

#### 2.4.4 Impedance variability

Impedance variability throughout the recording is a critical factor, potentially more so than the actual impedance value. To characterize this variability, impedance signals were processed using a 1-minute moving average method to mitigate potential noise interference and minimize variations in impedance that do not impact on analysis of voltage signal. Afterwards, the variable to quantify is the moving standard deviation computed for each electrode of each recording (29). Thirty-minute segments with a 50% overlap are utilized. Mean and standard deviation of the obtained values for each recording segment are computed to assess any statistical differences.

#### 2.4.5 Opinion survey

An opinion survey was conducted after each recording, to measure the satisfaction of the healthcare personnel with the new aCUP-E electrodes. The survey has been divided into 2 main sections to test the usability and the safety issues, as well as some comments from the nurses.

## 3 Results

### 3.1 Study population

The clinical trial has been carried out on 15 newborns that were admitted into the NICU of the HUSJR, classified in two groups: 4 at term and 11 preterm (between 32 and 36 gestational weeks). Only neonates with no suggestive signs of neurological damage, were eligible for the analysis. Table 2 shows the main characteristics of the neonates of the study (Gestational Age, Post Conceptional Age at the day of the study, Post Natal Age at the day of the study, gender and recording time).

**Table 2:** Sum up of the subjects of the study.

Case #	GA (weeks + days)	PCA (weeks + days)	PNA (days)	Preterm/term	Gender	aCUP-E hemisphere	Recording time
1	40 + 5	41 + 3	5	Term	F	Right	24 h 38 min
2	35	36	7	Preterm	M	Right	28 h 34 min
3	39 + 2	40	5	Term	F	Right	43 h 10 min
4	32 + 4	33 + 5	8	Preterm	M	Right	47 h 12 min
5	32 + 4	34	10	Preterm	F	Left	28 h 34 min
6	33 + 3	34 + 4	8	Preterm	F	Right	42 h 12 min
7	37 + 6	38 + 1	2	Term	F	Right	29 h 01 min
8	30 + 5	33 + 1	17	Preterm	F	Right	30 h 05 min
9	34 + 1	35 + 1	7	Preterm	M	Right	27 h 34 min
10	31 + 4	35	24	Preterm	M	Left	23 h 28 min
11	37 + 1	37 + 6	5	Term	M	Left	45 h 38 min
12	34 + 6	36 + 2	10	Preterm	M	Left	24 h 54 min
13	33 + 5	36 + 2	4	Preterm	F	Right	27 h 18 min
14	30 + 6	33 + 6	21	Preterm	M	Left	27 h 58 min
15	30 + 4	32 + 4	14	Preterm	M	Right	52 h 09 min

GA, gestational age; PCA, post conceptional age at the day of the study; PNA, post natal age at the day of the study.

### 3.2 Optimal and non-optimal brain activity

An algorithm was developed to detect non-cerebral brain activity, enabling the determination of optimal and non-optimal brain activity segments. Non-optimal segments indicate the presence of artifacts, while optimal segments suggest exclusive detection of brain activity.

**Figure 6:**
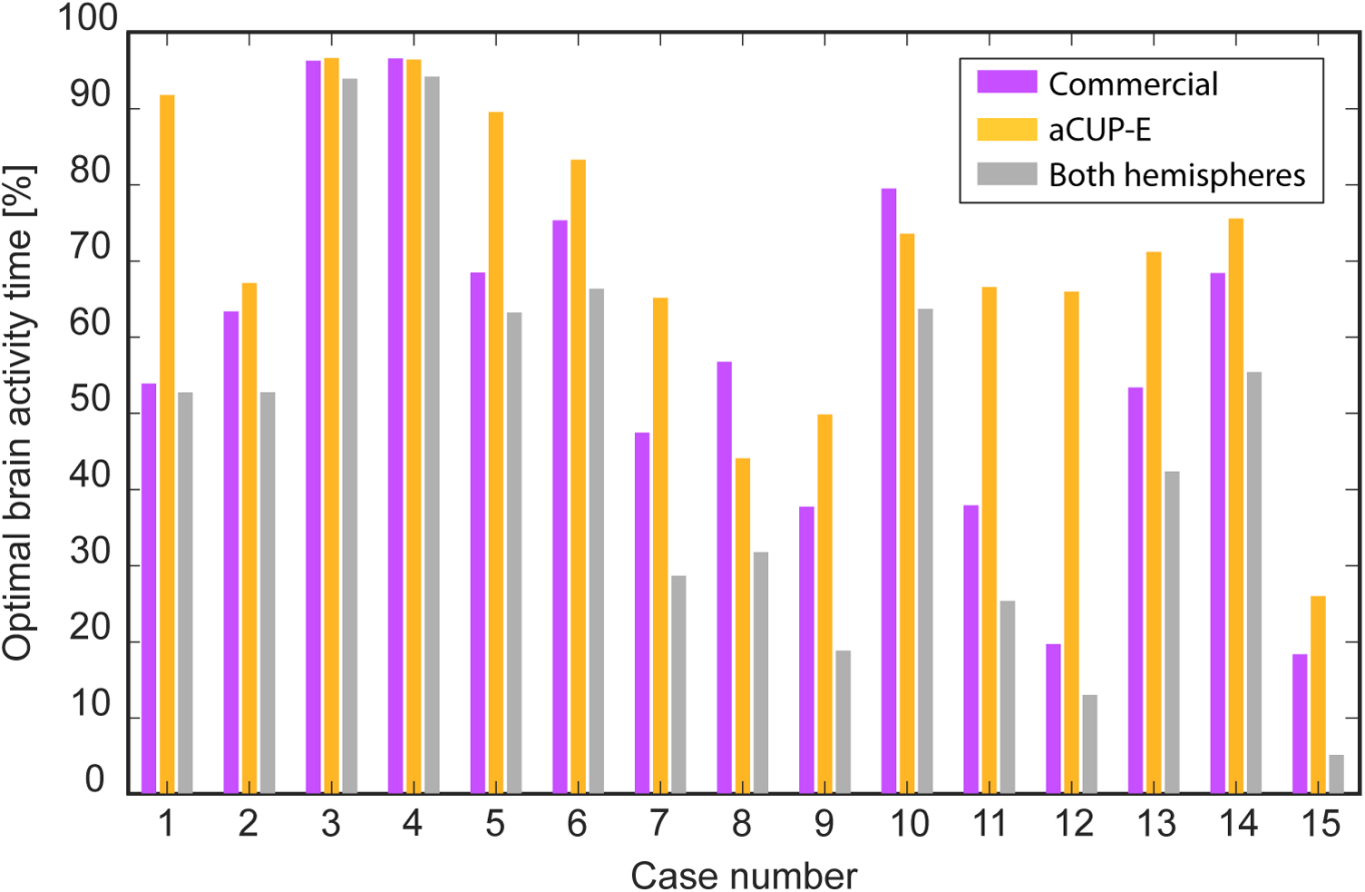
Summary of the optimal brain activity captured for each electrode along the different cases. (Purple) Commercial electrodes, (Orange) aCUP-E, (Grey) Both electrodes optimally recording simultaneously.

These results are presented for Neuroline 720 (commercial), aCUP-E, and both hemispheres, with optimal brain activity defined as simultaneous optimal activity in both electrodes (Figure 6). This feature is crucial for continued analysis as it allows assessment of signal capture in long artifact-free segments to evaluate electrode response consistency.

Overall, it is observed that commercial electrodes tend to limit the duration of analysis where both hemispheres can be simultaneously assessed. Exceptions to this trend (e.g., cases 8 and 10) can be attributed to challenging conditions such as incubator use and thick hair, leading to poor electrode performance (e.g., inadequate adhesion and high spike counts).

Variations in the percentage of optimal time between recordings are influenced by individual case characteristics. While difficulties experienced by some subjects may impact aCUP-E performance, they influence commercial electrodes as well (e.g., case 15). There are instances where these challenges disproportionately affect current clinical electrodes (e.g., cases 1, 5, and 7).

### 3.3 Reliable signal capture

Bland-Altman plots have been used to show how, in fragments where both electrodes have been optimally recording, both capture a similar signal. Since aEEG is obtained from a bipolar montage, the positioning as well as the impedance of each of the electrodes have effects on the voltage (27), even that the signal is still interpretable by the clinician.

**Figure 7:**
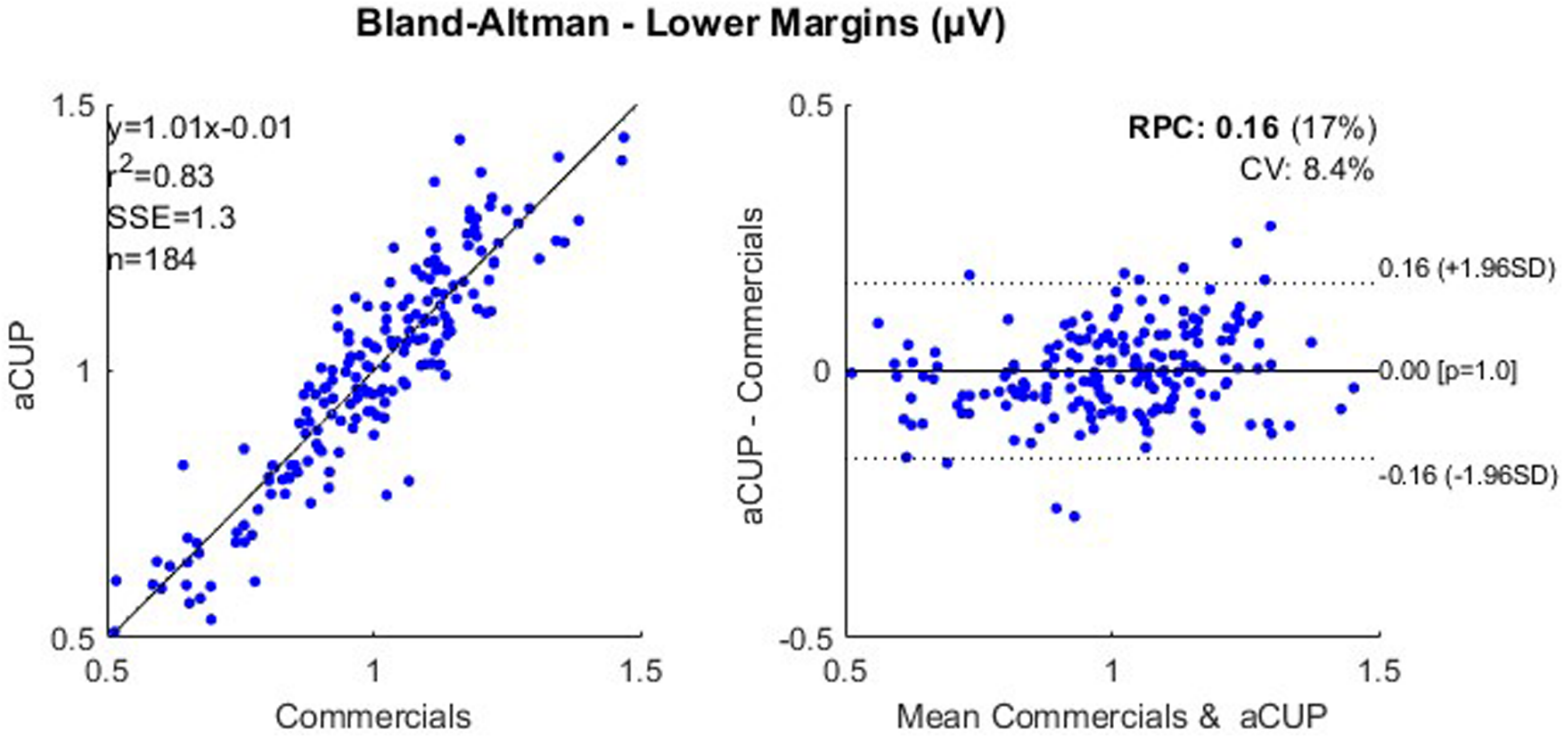
Correlation plot (left) and bland-altman plot (right) for the lower margin values of optimally recorded segments of 2 h from cases 1, 2, 3 and 4; where RPC is the reproducibility coefficient and CV is the coefficient of variation. RPC. reproducibility coefficient; CV, coefficient of variation.

**Figure 8:**
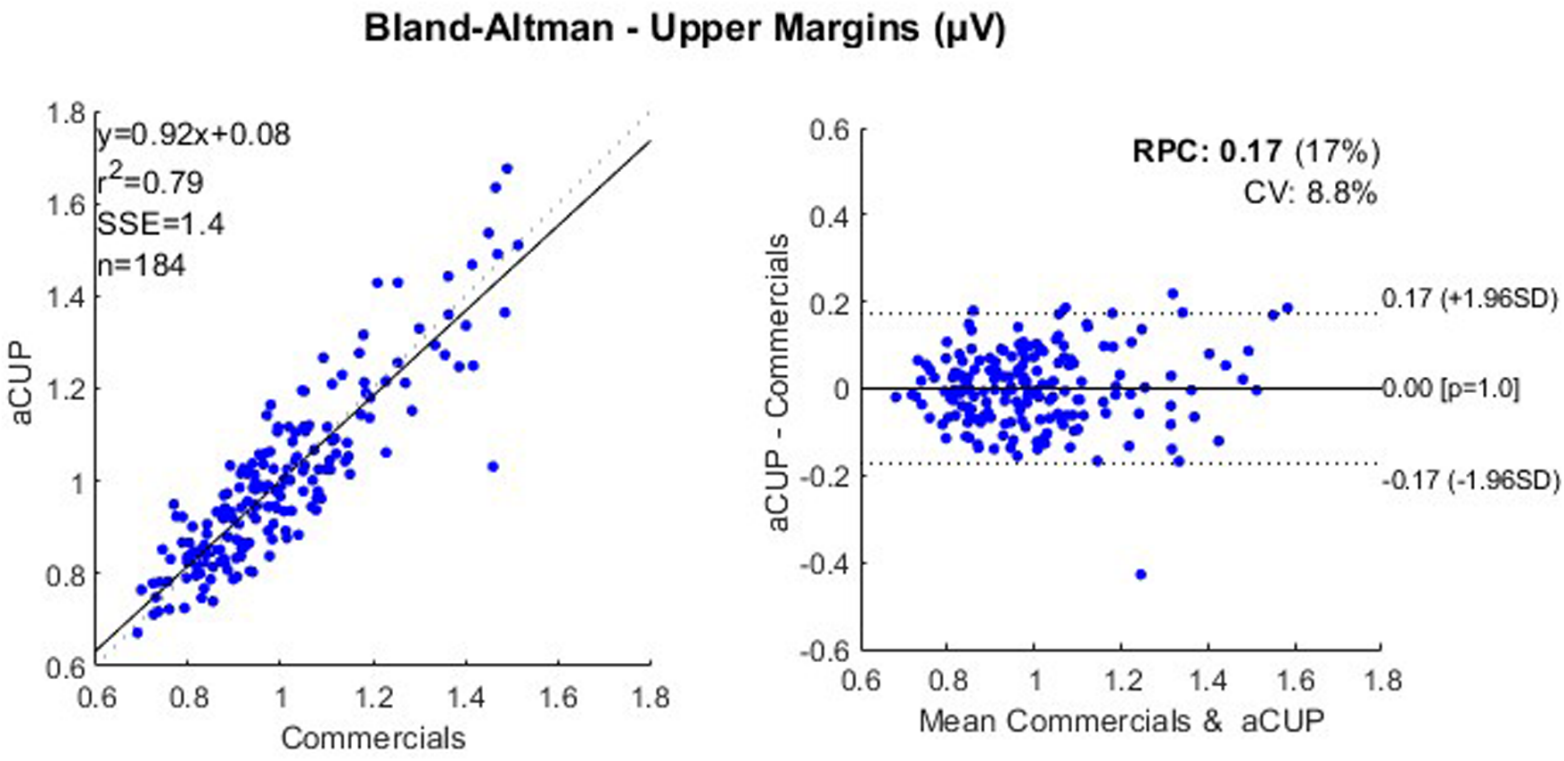
Correlation plot (left) and bland-altman plot (right) for the upper margin values of optimally recorded segments of 2 h from cases 1, 2, 3 and 4; where RPC is the reproducibility coefficient and CV is the coefficient of variation. RPC, reproducibility coefficient; CV, coefficient of variation.

To correct these errors, the feature shown in the following plot is the quotient of the margin value and the mean margin value for that segment (2 h). So, as a result we obtain 2 Bland-Altman (Figures 7, 8), one for each margin, showing how the capture of the signals resembles, only in the optimally recorded segments. With these results, it can be stated that the capture of the signals in ideal segments is highly correlated for both electrodes.

**Figure 9:**
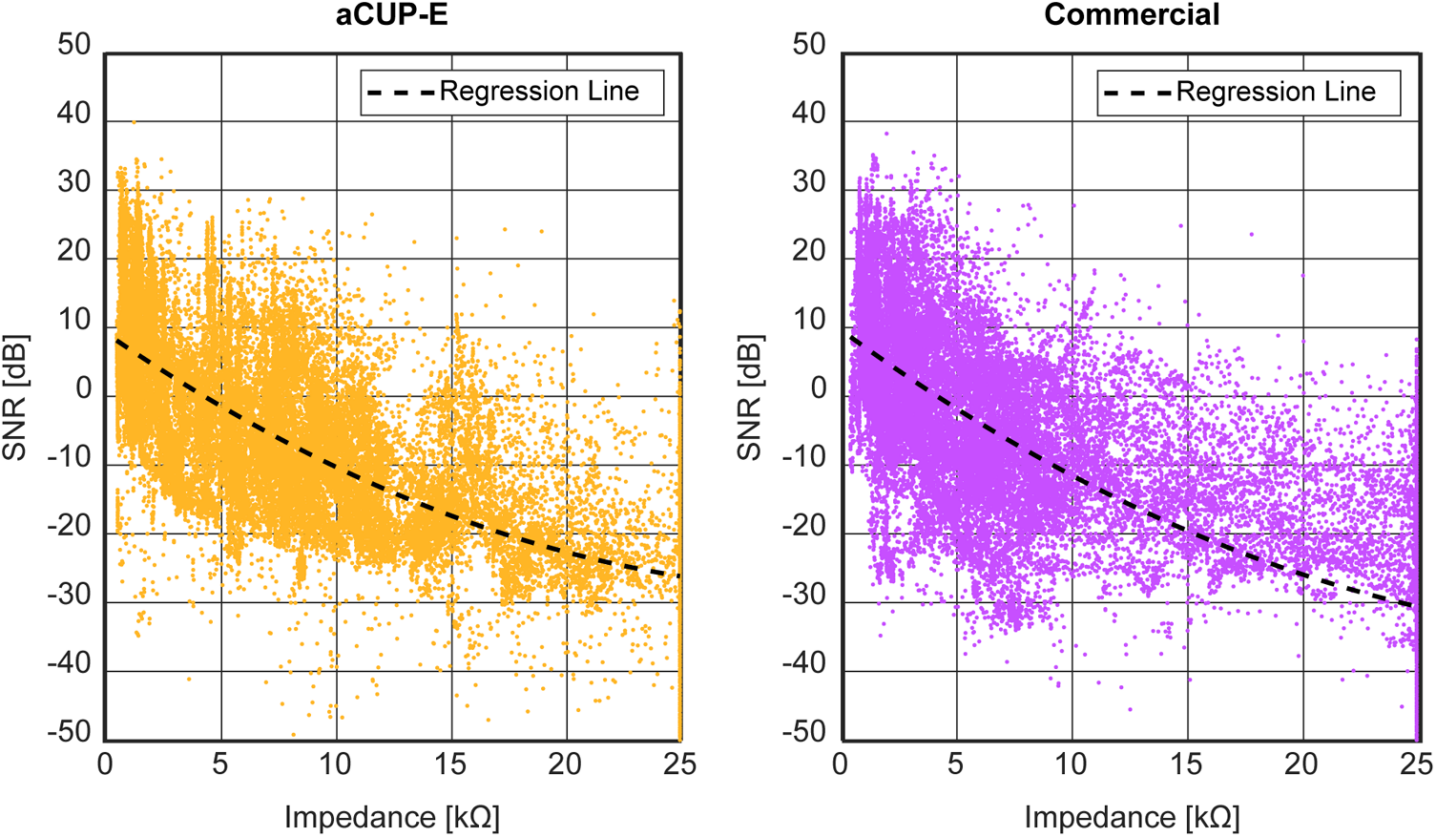
Signal to noise ratio (SNR) captured for aCUP-E (left) and commercial (right) electrodes in dB at different impedances (kΩ). The 15 recordings are included in the graph, and each point represents a 1 min segment of one of them. Black dashed line represents the regression line (2nd order polynomial) from the data, which estimates the natural behaviour of the electrode.

While the aforementioned analysis confirms nearly identical voltage capture by both electrodes, it is restricted to optimal time frames. However, susceptibility analysis (Figure 9) assessing SNR demonstrates that both electrodes have the same signal capture, making aCUP-E electrodes reliable. This curve has been fitted by means of a polynomial, an exponential and a potential equation, always obtaining a similar fitting coefficient (R^2^ adjusted and RMSE), with differences of less than 3%.

Noise susceptibility is a characteristic of the electrode plate, where the contact is a crucial factor (30). Therefore, the similarities found between electrodes could be expected, since the sensor material is not a differential factor of aCUP-E. The previous regressions would be a modelling of what is the behaviour of an Ag/AgCl electrode when performing electroencephalography.

### 3.4 Electrode survival

This section examines all recordings, considering instances where a cessation of functioning is detected, leading to electrode replacement in any case. Main factors are electrode detachment and gel drying but other factors may have contributed to electrode failures. For instance, longer manipulation times or parental desires to hold their children, resulting in the inadvertent disconnection of electrodes from the monitor, leading to increased impedances across all electrodes simultaneously. These cases were individually assessed based on nurse observations documented in the monitor, with each impedance curve interpreted to discern causative factors. In addition, in some instances, the application of electroconductive gel to the aCUP-E electrodes every 8 h was overlooked, resulting in a cessation of functioning. However, this lapse corresponds to a breach of the protocol, indicating non-compliance with guidelines.

**Figure 10:**
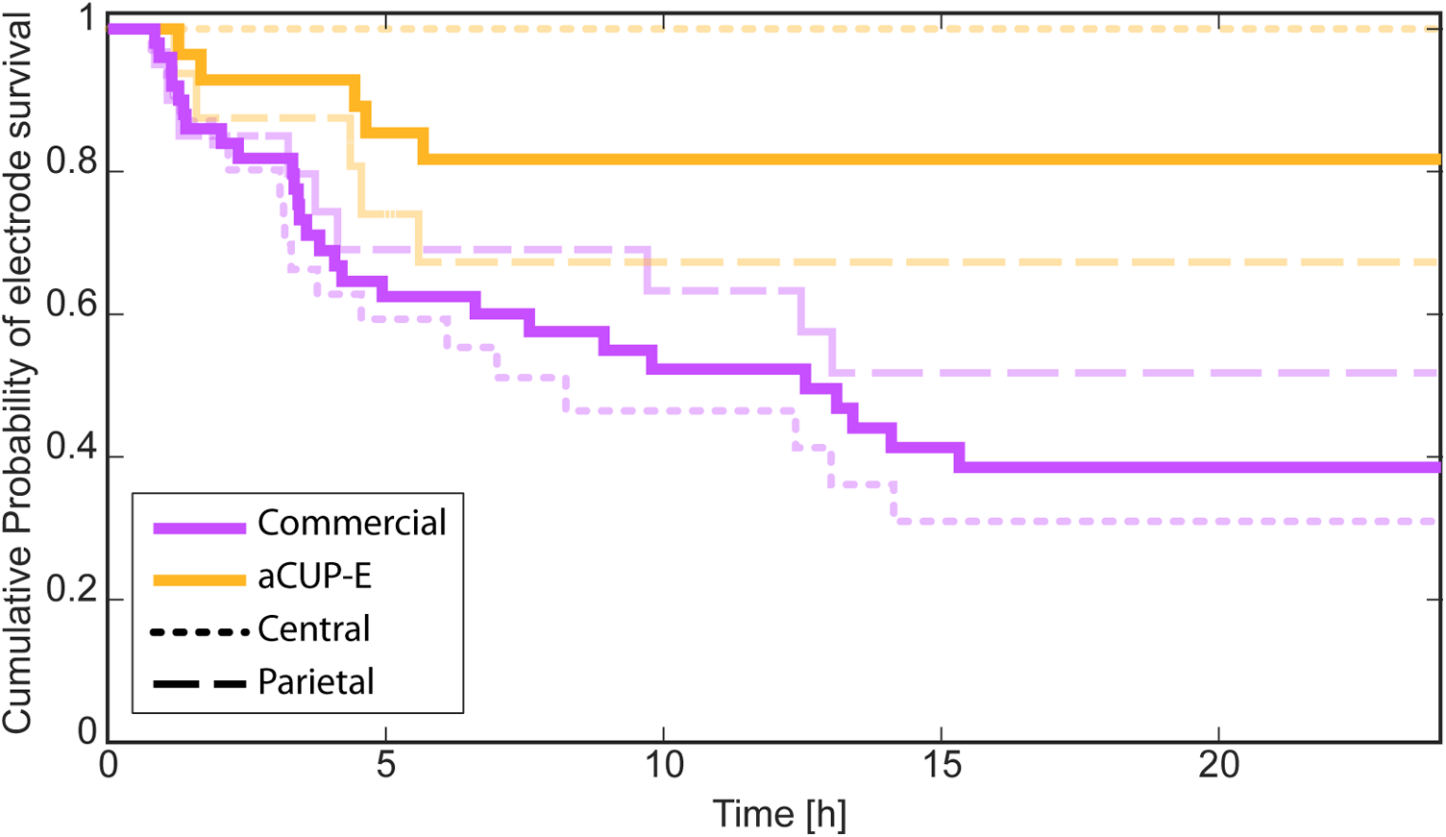
Corrected survival analysis (Kaplan-Meier curve) in which manipulations and breaches of the protocol are not considered as a cease of the functioning. Results obtained considering all the electrodes together (Tarone-Ware: X=11.38, p=7.42e−4), and separately analysing the electrodes on the central (C3-C4) positions (Tarone-Ware: X=14.87, p=1.15e−4), and on the parietal (P3-P4) positions (Tarone-Ware: X=0.70,p=0.40).

None of the central aCUP-E electrodes have failed in the 24 h of recording (Tarone-Ware: X=14.87, p=1.15e−4), while in the commercial ones less than the 40% have survived the first 24 h (Figure 10). Regarding the parietal, both still show a similar curve along the recording. However, there is a greater difference in survival at in higher times, where almost 65% of aCUP-E have survived, while only 50% of commercials.

In the study performed by Ters et al. (14) on electrode survival, a comparison between hydrogel gel electrodes and gold cup electrodes was performed. Results indicated that at the 24-hour mark, hydrogel electrodes exhibited similar survival rates to liquid gel electrodes (less than 40%), while gold cup electrodes fared even worse. This further supports the validity of our findings and underscores aCUP-E as the electrode with the highest probability of survival.

### 3.5 Impedance variability

From the obtained value of the moving standard deviation for the segments of each recording, the mean value for each subject is computed to avoid giving more importance to longer recordings but considering all the segments. In this section the statistical test is one-sided since the alternative hypothesis is that aCUP-E is more stable than the commercials.

**Figure 11:**
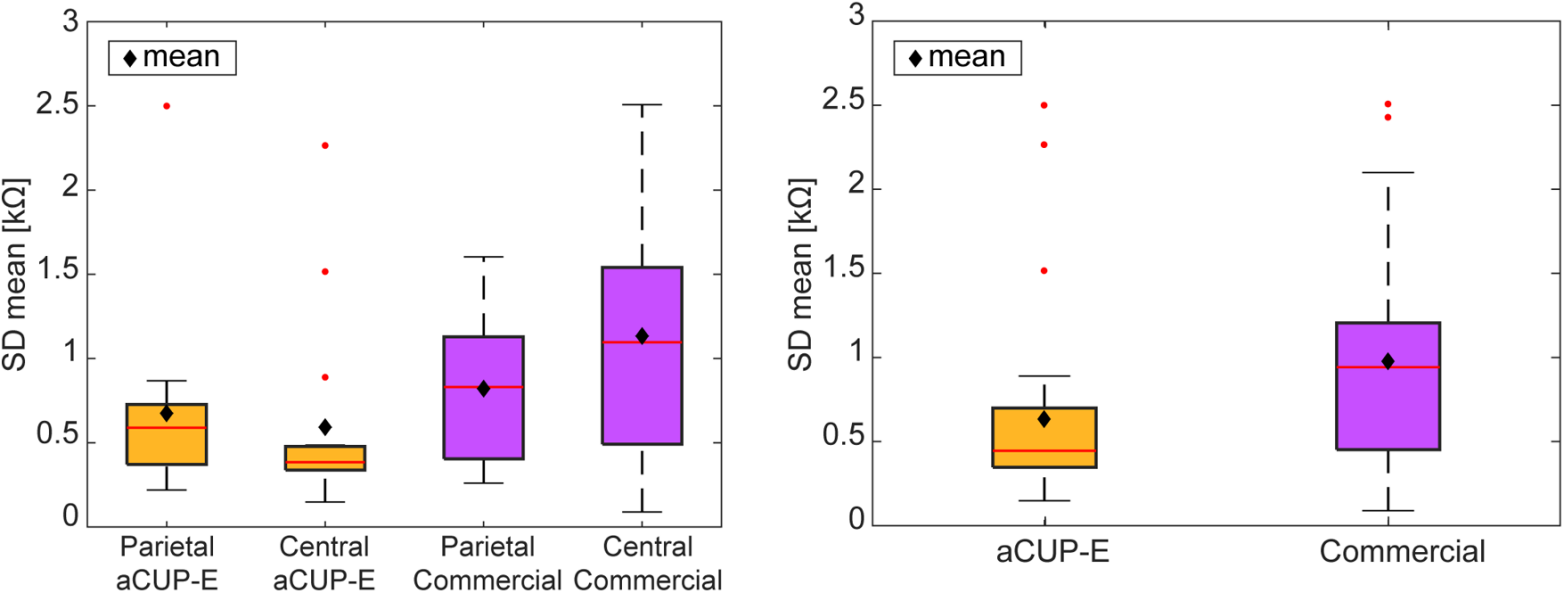
Average of the standard deviation of each segment (30 min) for each record. Impedance variability among segments (SD) of each recording. (Left) Mean variability value of each electrode type and position for every recording. (Right) Variability value of each electrode type (mean between locations) for every recording. Red points are outliers while black diamonds are the mean for all patients.

Individual position evaluations revealed that central aCUP-E electrodes outperformed other central electrodes significantly (p=0.017). For the parietal electrodes, the results still show that aCUP-E performs better (p=0.06). Figure 11 provides a visual comparison of aCUP-E performance in terms of impedance stability. Even so, when doing an overall assessment without distinguishing between positions, commercials electrodes are less stable than aCUP-E (p=0.0043).

Overall, the analysis demonstrates that aCUP-E electrodes exhibit superior impedance stability, addressing a significant concern in aEEG. Previous research has highlighted the challenges associated with maintaining stable impedance levels using hydrogel electrodes and emphasized the importance of proper skin preparation (11). Therefore, aCUP-E electrodes present a promising non-invasive alternative to improve this feature.

### 3.6 Opinion survey

#### 3.6.1 Ease of electrode placement and removal

In terms of ease of placement, aCUP-E electrodes are favoured by NICU healthcare staff due to their stable adhesion, facilitating smooth placement and improved handling of neonates. Moreover, it allows the use of the kangaroo care method which is extensively used in the NICU to promote skin-to-skin contact with the parents, having numerous benefits for the preterm infants.

Conversely, commercial electrodes exhibit inferior adherence, leading to challenging placement and an uncomfortable handling of the neonate, because they may detach. While this results in easier removal, aCUP-E electrodes do not require difficult efforts for detachment once the recording is finished.

#### 3.6.2 Skin safety

To assess skin safety issues, three characteristics have been checked: dryness, erythema (redness) and break-down, utilizing the Neonatal Skin Condition Score (NSCS) scale. None of the patients had significant skin issues.

Commercial electrodes are skin safe for the neonate because of their low adhesion capacity, but their lack of fixation requires frequent changes, increasing potential skin safety issues due to skin preparation.

Overall, aCUP-E electrodes improve skin safety because of the reduction of skin preparation procedures (not being replaced). The highest adhesion of aCUP-E may require (as a function of subject's skin condition and hair quantity) the use of adhesive removal to simplify the procedure. However, it is also used in other type of electrodes and does not compromise the fragile skin of infants. Additionally, aCUP-E electrodes are translucent, allowing for cutaneous observation.

## 4 Conclusions

The results of the present study show that aCUP-E electrodes exhibit better adhesion and higher impedance stability compared to current clinical practice electrodes. This translates to improved signal quality and prolonged electrode lifespan, enhancing the interpretation of the aEEG signal for improved newborn diagnosis and treatment. These outcomes underscore the deficiencies of existing electrodes and emphasize the potential of new specific electrodes for newborns such aCUP-E as a better solution. aCUP-E records larger optimal brain activity compared to the commercial ones, indicating reduced sensitivity to various artifacts. This suggests the potential for expanding aEEG utilization in rapidly diagnosing and treating neonates through extended recording periods.

aCUP-E performed significantly better in terms of impedance stability and electrode life because of their improved adhesion and gel replacement ability. Dramatic differences were achieved for central positions, while parietal locations show minor differences. Reduced parietal differences may be because of the position of the wire exiting vertically or the higher body may make them more vulnerable to friction forces with the mattress. However, aCUP-E exhibits comparable signal capture as commercial electrodes across the different impedance levels, showing their equivalence to current clinical electrodes. Notably, lower impedance variability has been observed compared to commercial electrodes.

Survey responses from NICU staff indicate that aCUP-E electrodes alleviate workload burdens by simplifying impedance level monitoring for optimal recordings. Moreover, the aCUP-E adaptations allow for the kangaroo care method which promotes skin-to-skin contact and supporting the well-being of premature neonates.

Results may allow improvements to other electrodes developed and future versions of aCUP-E electrode to even better reduce artifacts and increase electrode life by enhancing artifact reduction and electrode longevity. The study highlights the significance of these results in clinical practice, confirming previous laboratory findings and paving the way for high-quality aEEG recordings in NICUs worldwide. Ultimately, enhanced electrode performance will enable neonatologists to diagnose and treat newborns more efficiently in the future.

It is important to acknowledge several limitations of the current study. First, the sample size (15 patients) limits the generalizability of the findings and may introduce bias. Future studies with larger patient cohorts are essential to validate the results. Second, the study focused on a recording duration of 24 h. This monitoring period may not fully capture the long-term performance and reliability of the aCUP-E electrodes, especially in clinical situations requiring extended monitoring (e.g., therapeutic hypothermia). Moreover, due to issues encountered with commercial electrodes, it has been challenging to obtain longer, uninterrupted recordings, which restricts our ability to make comprehensive comparisons over extended periods.

Additionally, the study was conducted at a single center, which may limit the applicability of the results to other clinical settings. We recognize the importance of evaluating the device's performance in more immature premature infants with a lower gestational age and assessing the effects on their more delicate skin. Future research will address these limitations through multicentric trials with a neurologically non-healthy neonates’ population, extended monitoring periods and the evaluation of results in younger premature infants.

## Data Availability

The datasets presented in this article are not readily available because the data supporting the findings of this study are not publicly available due to patient confidentiality and participant privacy concerns, as well as ongoing intellectual property protection (patent pending). However, data can be made available upon reasonable request to the corresponding author, contingent upon the necessary permissions from third parties, including the ethical committee and the patent proprietors. Researchers seeking access to the data should provide a detailed request outlining the purpose and scope of their proposed use and agree to abide by all applicable confidentiality and data protection protocols. Requests to access the datasets should be directed to a.fabregat@urv.cat.

## Footnotes

^1^If at the *refilling step* commercial electrodes are still adhered, they should remain untouched since the manufacturer does not ensure a good adhesion after removing them, what would be necessary to add more gel.
